# Clinical characteristics, prognosis, and achievement of transplant in adolescents and adult patients with Philadelphia chromosome-negative acute lymphoblastic leukemia in Argentina

**DOI:** 10.1016/j.htct.2026.106530

**Published:** 2026-09-07

**Authors:** María Manuela Clavijo, Juan Ignacio Ruiz, Luciana Ferrari, Alberto Gimenez Conca, Agostina Delorenzi, Leticia Rapan, Nicolas Cazap, María Marta Rivas, Eliana Moya, Lucía Agamennoni, Celina Guadalupe Vega, Julian Freue, Maria Eugenia Funes, Hernan Daniel Dick, Mercedes Mac Mullen, Alicia Navickas, Isolda Fernandez, Natalia Carnelutto, Claudia Vicente, María Moirano, Ana Lisa Basquiera, Carolina Belli

**Affiliations:** a Department of Hematology, Hospital Alemán, Ciudad Autónoma de Buenos Aires, Argentina; b Department of Health Services Research, The University of Texas MD Anderson Cancer Center, Houston, Texas, United States; c Department of Hematology, Fundaleu, Ciudad Autónoma de Buenos Aires, Argentina; d Department of Hematology, Hospital Italiano, Ciudad Autónoma de Buenos Aires, Argentina; e Department of Hematology, Hospital Austral, Pilar (Buenos Aires), Argentina; f Department of Hematology, Sanatorio Sagrado Corazón, Ciudad Autónoma de Buenos Aires, Argentina; g Department of Hematology, Hospital CEMIC, Ciudad Autónoma de Buenos Aires, Argentina; h Department of Hematology, Hospital Central de Mendoza, Mendoza (Mendoza), Argentina; i Department of Hematology, Hospital El Cruce, Florencio Varela (Buenos Aires), Argentina; j Pfizer SRL, Villa Adelina (Buenos Aires), Argentina; k Department of Hematology, Sanatorio Anchorena, Ciudad Autónoma de Buenos Aires, Argentina; l Department of Hematology, Sanatorio Británico, Rosario (Santa Fe), Argentina; m Department of Hematology, Hospital Italiano de La Plata, La Plata (Buenos Aires), Argentina; n Department of Hematology, Hospital de Clínicas José de San Martín, Ciudad Autónoma de Buenos Aires, Argentina; o Department of Hematology, Hospital San Martín de La Plata, La Plata (Buenos Aires), Argentina; p Department of Hematology and Oncology, Hospital Privado Universitario de Córdoba, Córdoba (Córdoba), Instituto Universitario de Ciencias Biomédicas de Córdoba (IUCBC), Argentina; q Department of Hematology, Instituto de Medicina Experimental (IMEX-CONICET), Ciudad Autónoma de Buenos Aires, Argentina

**Keywords:** Acute lymphoblastic leukemia, Allogeneic bone marrow transplantation, Hematological malignancies

## Abstract

**Introduction:**

Data on clinical outcomes and allogeneic stem cell transplantation for Latin American Philadelphia chromosome-negative acute lymphoblastic leukemia patients are limited.

This study aimed to characterize this population and identify factors influencing overall survival among adolescents and adults with this malignancy in Argentina. Additionally, the study examined the predictors of proceeding to first-line allogeneic stem cell transplantation in this population.

**Methods:**

This retrospective cohort study included patients diagnosed at 15 centers between 2014 and 2022. Descriptive, univariate, and multivariate analyses were conducted.

**Results:**

Three hundred and five patients with a median age of 34 years were included. Median overall survival was 42 months. Treatment at public healthcare institutions (hazard ratio: 1.61), central nervous system involvement (hazard ratio: 1.56), high-risk status defined by the receipt of Berlin-Frankfurt-Münster-like or hyperfractionated cyclophosphamide, vincristine, doxorubicin, and dexamethasone (hyper-CVAD) regimens (hazard ratio: 1.89), and older age (hazard ratio: 1.02) were independently associated with a worse outcome. In a subgroup analysis of 199 patients with an indication and eligibility to receive allogeneic stem cell transplantation, 20.1% underwent the procedure. Receiving treatment at public institutions was associated with a 65% lower likelihood of undergoing transplantation (p-value = 0.013), an effect that remained consistent when death or relapse was accounted for as a competing event (Subdistribution Hazard Ratio: 0.28).

**Conclusions:**

In this acute lymphoblastic leukemia Argentinean cohort, overall survival was influenced by classical predictors of a worse outcome and by healthcare disparities. Receiving treatment at public institutions adversely diminished access to first-line allogeneic stem cell transplantation, which may account for the poorer outcome.

## Introduction

Acute lymphoblastic leukemia (ALL) is a heterogeneous neoplasm characterized by a proliferation of immature lymphoid cells. It is most common in childhood with a smaller second peak at around 50 years. The estimated overall incidence is 1.28–1.70 per 100,000 persons annually in the United States [1,2] and, although its incidence is unknown in Argentina, some studies have reported an increased frequency in South American countries [[2], [3], [4], [5]].

Five-year overall survival (OS) rates consistently decrease with age. Considering chemotherapy-based treatment regimens, excluding the use of novel therapeutic agents, reported OS is approximately 93% for children, 74% for adolescents, 59% for young adults (20–39 years), 43% for middle-aged adults (40–59 years) and consistently below 30% for those older than 59 years [1]. Prognostic heterogeneity is determined by clinical characteristics, cytogenetic and molecular findings, and the response to treatment, including the measurable residual disease (MRD) status [6]. Different therapeutic protocols stratify patients into risk-adapted treatment arms of varying intensity, primarily based on the presence of high-risk features [6]. Despite advances in first-line therapies, one-third of standard-risk and up to two-thirds of high-risk patients relapse, which represents a major therapeutic challenge [6]. In general, patients classified as high-risk at diagnosis, those with positive MRD after first complete remission, and those who relapsed and achieved a second complete remission, are considered candidates for allogeneic hematopoietic stem cell transplantation (allo-HSCT) [7,8].

Basic treatments appear to be generally guaranteed according to surveys among hematologists from Latin American Societies. However, considerable heterogeneity has been described regarding access to adequate diagnostic tools, high-cost therapies and allo-HSCT, mainly depending on whether patients have public or private health insurance [9].

Some reports have described particular aspects regarding clinical and prognostic characteristics, therapeutic approaches, and access or outcomes related to allo-HSCT in selected aged cohorts of ALL patients across Latin America [4,5,[10], [11], [12], [13], [14], [15]]. Since previous studies in Argentina have focused on participants up to young adulthood [12,14], the aim of this study was to provide insights into demographic characteristics and outcomes of adolescent and adult patients with ALL in Argentina. The second purpose was to identify factors influencing the access to first-line HSCT.

## Methods

### Study population and design

A multicenter retrospective cohort study was conducted including adolescents (aged ≥15 years) and adults diagnosed with Philadelphia chromosome-negative (Ph- negative) ALL between 2014 and 2022.

Member healthcare centers of the “Grupo Argentino de Leucemias Agudas (GALA)”, a part of the Argentinean Society of Hematology (SAH), were invited to participate. Clinical data were collected from medical records at each participating institution, including demographic and clinical characteristics, date of ALL diagnosis, treatment protocol, HSCT, and dates of relapse or death. Treatment regimens were classified as either Berlin-Frankfurt-Munster (BFM)-like or hyper fractionated cyclophosphamide, vincristine, doxorubicin, and dexamethasone (hyper-CVAD) (Supplementary material, Appendix 1). Lineage (B or T) was determined by immunophenotyping using multiparametric flow cytometry (MFC). Cytogenetic and/or fluorescence in situ hybridization (FISH) evaluation analyses at diagnosis included *BCR::ABL1* testing. Central nervous system (CNS) involvement was defined by cerebrospinal fluid (CSF) analysis using MFC, and occasionally by imaging when clinically indicated. Patients lacking essential information were excluded. The study protocol was initially approved by the Ethics Committee of Hospital Alemán (CEIHA, CODE 1186, v5.0 [18 November 2020]), and, subsequently, by the local ethics committees of each participating institution.

### Definition of response criteria

Morphological complete remission (MCR) at Day 33 was defined as a bone marrow smear with normal or slightly decreased cellularity, no localized leukemic infiltrates or masses, and no leukemic cells in the CSF. Evaluation of MRD was performed on fresh bone marrow aspirates via 8-color MFC with a sensitivity of 10^−4^. Late response was defined as an MRD ≤0.01% attained by Day 70–78 or at three months according to the treatment protocol, whereas refractoriness was based on lack of response or persistence of positive MRD after the first consolidation block [12].

### Statistical analysis

A descriptive analysis was performed to characterize the study population. Continuous variables were summarized as median and interquartile range (IQR) or their corresponding 95% confidence interval (95% CI). Categorical variables were reported as percentages or frequencies. OS and time to HSCT were estimated using the Kaplan–Meier method. Time-to-event endpoints were calculated from the date of diagnosis to death, last follow up, censoring at the time of HSCT in first remission or at the date of the procedure. Cox proportional hazard regression models were fitted for univariate and multivariate analyses, and hazard ratios (HR) with 95% CI were reported to adjust for potential confounders. For time-to-HSCT, a Fine-Gray competing risk model was used considering death or relapse as competing risk events. Cumulative-risk rate was estimated using the cumulative incidence function.

Variables with a p-value ≤0.20 in univariate analysis or deemed clinically relevant were included in multivariate models when a p-value <0.05 was considered statically significant. Analyses were performed using Rstudio (version 2023.06.0 + 421).

## Results

### Patient characteristics and responses

Data were obtained from 15 healthcare centers, including four public institutions (27.0%) and 11 private centers (73.0%). Ten centers (67.0%) were located in the Buenos Aires Metropolitan Area, and five were in other regions of Argentina (33%).

The demographic and clinical characteristics of the cohort are detailed in Table 1. Among the 305 participants, 62.0% were male, and the median age was 34 years. At diagnosis, the median white blood cell count was 12.25×10^9^/L (IQR: 34.00–50.00×10^9^/L), the median platelet count was 41.00×10^9^/L (IQR: 22.00–88.00×10^9^/L), the median hemoglobin level was 8.8 g/dL (IQR: 7.0–10.5 g/dL), and the median percentage of blast cells was 68% (IQR: 30%−88%). Extramedullary involvement was observed in 35.4%, with CNS involvement in nearly half of these cases (19.3%). The most frequent immunophenotype observed was common B-cell ALL (60.7%). Although all patients were tested for *BCR::ABL1* molecular rearrangement, karyotype and molecular findings (described in Table 1) were unavailable for 85 patients (27.9%), including 45/121 (33.9%) from public and 40/184 (21.7%) from private institutions (p = 0.003) (Table S2).

**Table 1:** Patient characteristics (n = 305).

Characteristic	n (%)
Gender - Female - Male	116 (38.0) 189 (62.0)
Age (years) - Median age (IQR) - 15 – 40 - 41 – 60 - >60	34 (25–49) 217 (71.1) 65 (21.3) 23 (7.5)
Healthcare center for leukemia treatment - Public - Private	121 (39.7) 184 (60.3)
Location of the institution for leukemia treatment - Ciudad Autónoma de Buenos Aires - Buenos Aires province - Rest of the country	126 (41.3) 133 (43.6) 46 (15.1)
Charlson’s comorbidity index - <2 - ≥2 - Not reported	250 (82.0) 42 (13.8) 13 (4.3)
Immunophenotype Immunophenotype B - Common B - Pre-B - Pro-B - Unclassified B Immunophenotype T - Pre-T (II) - Early T - Pro-T - Cortical T (III) - Mature T (IV) - Unspecified T Biphenotypic Not reported	247 (81.0) 185 (60.7) 30 (9.8) 24 (7.9) 8 (2.6) 55 (18.0) 15 (4.9) 2 (0.7) 5 (1.6) 19 (6.2) 3 (1.0) 11 (3.6) 2 (0.7) 1 (0.3%)
CD20 frequency* - Negative - Positive - Not reported	169 (68.4) 73 (29.6) 5 (2.0)
Risk stratification - High risk - Standard risk - Not reported	181 (59.3) 113 (37.0) 11 (3.6)
Karyotype/molecular findings - Normal karyotype - Hyperdiploid - t(1;19) - Hypodiploid - 11q23/*KMT2A* rearrangements - Complex karyotypes - Others - No data	122 (40.0) 8 (2.6) 6 (2.0) 6 (2.0) 11 (3.6) 24 (7.9) 43 (14.1) 85 (27.9)
Extramedullary involvement - CNS involvement	116 (35.4) 59 (19.3)
Treatment - BFM-like regimen - Hyper-CVAD - Not reported	276 (90.5) 18 (5.9) 11 (3.6)

CNS: central nervous system; BFM: Berlin-Frankfurt-Munster; hyper-CVAD: hyper fractionated cyclophosphamide, vincristine, doxorubicin, and dexamethasone.

⁎ CD20 among immunophenotype B-cell acute lymphoblastic leukemia (n = 247).

Regarding treatment, BFM-like regimens were administered in 90.5% of the cases: 29 patients were treated according to the PETHEMA (Spanish Program for the Treatment of Malignant Blood Diseases) algorithms and 247 as outlined by GATLA (Grupo de Tratamiento de Hemopatias Malignas, Argentina). Eighteen (5.9%) patients were administered a hyper-CVAD scheme. Notably, among 73 patients with CD20^+^ B-lineage ALL (29.6% of 247 B-lineage cases), 33 (45%) received Rituximab without significant differences between private and public centers (54.3%vs. 36.8%; p = 0.155) (Table S2).

Among the 305 evaluable patients, 80.1% achieved MCR: 216 (70.8%) responded by Day 33, 31 (10.2%) were late responders, and 34 (11.1%) were refractory. During follow-up, 90 (29.5%) patients relapsed. According to each protocol (Supplementary material: Appendix 1), 59.3% were classified as high-risk and 37.0% as standard-risk, which reflects a predominance of high-risk features within the cohort.

### Overall survival

With a median follow-up from diagnosis of 27.8 months (IQR: 8–39 months) for the overall cohort, 142 (46.5%) died. Early deaths within the first three months occurred in 30 patients (9.8%): 21 deaths were due to infection, one to toxicity, three to major bleeding, one to progression, and four to other causes. The cause of death was specified in 101 cases: 48 from public and 53 from private centers (p = 0.101) (Supplementary Table S2). The two-year OS was 54.9% (95% CI: 48.9–61.6), with a median of 42.0 months (95% CI: 29.0–67.0 - Figure S1). On censoring at the time of HSCT procedure, the median OS decreased to 30.9 months (95% CI: 23.0–48.5). Univariate analyses are detailed in Supplementary Table S1.

**Figure 1:**
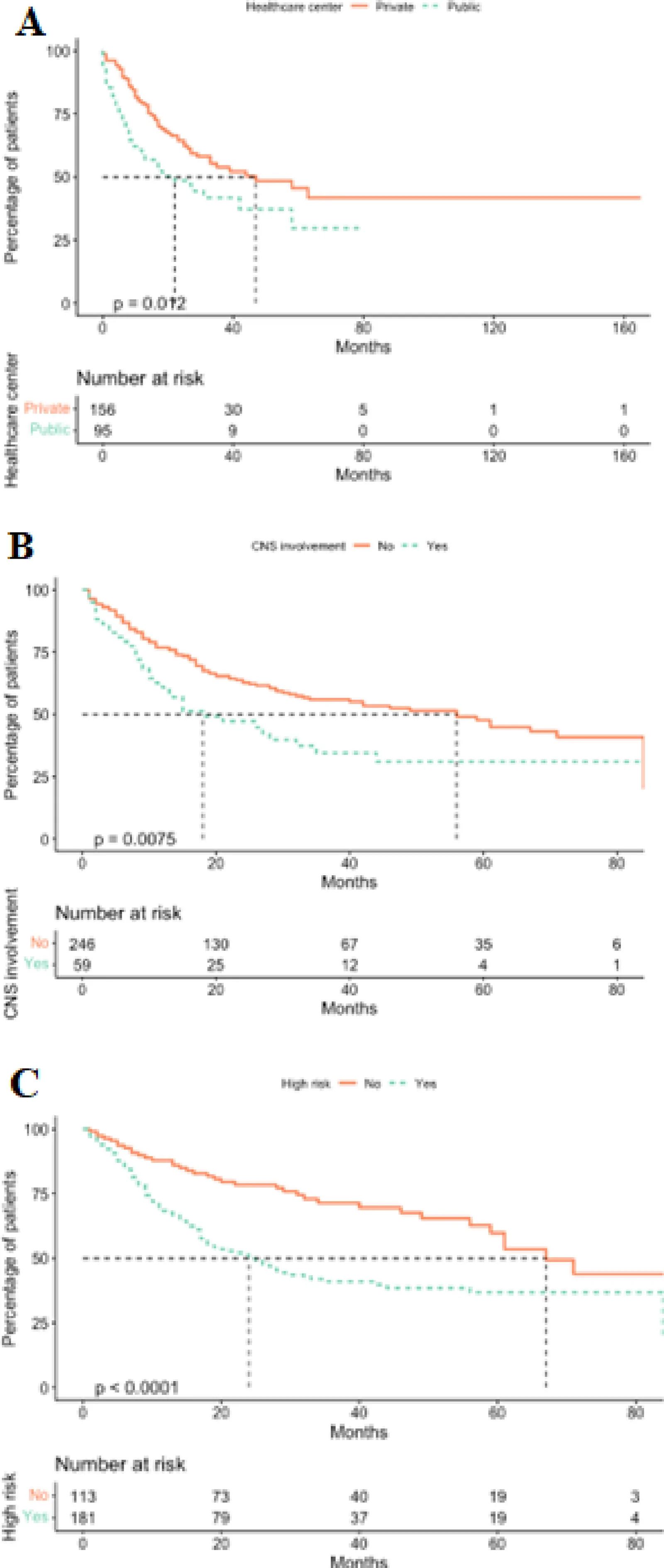
Kaplan-Meier plots and Log-Rank test - Overall survival according to A) Healthcare center; B) Central nervous system (CNS) involvement; C) Risk assessment.

The multivariate analysis (Table 2) showed that variables significantly associated with worse OS were treatment in public healthcare centers (HR: 1.61), CNS involvement (HR: 1.56), high-risk status (HR: 1.89) and older age (HR: 1.02). The respective Kaplan-Meier curves are shown in Figure 1. Baseline characteristics differed between private and public healthcare centers: patients from private centers were older (median age 30.2 vs. 24.5; p < 0.001) and had a higher proportion of a Charlson’s comorbidity index ≥2 (13.6%vs. 5.0%; p = 0.019) (Supplementary Table S2).

**Table 2:** Overall survival from diagnosis (Cox regression multivariate analysis).

Covariate	HR (95% CI)	p-value
Public (vs. private)	1.61 (1.10–2.35)	**0.015**
Male (vs. female)	1.31 (0.90–1.90)	0.164
Charlson’s comorbidity index*	1.07 (0.84–1.38)	0.570
CNS involvement (vs. no involvement)	1.56 (1.02–2.38)	**0.040**
BFM (vs. hyper-CVAD)	1.24 (0.59–2.60)	0.579
High risk (vs. non-high risk)	1.89 (1.26–2.84)	**0.002**
Age*	1.02 (1.01–1.03)	**0.003**

95% CI: 95% confidence interval; HR: hazard ratio; CNS: central nervous system; BFM: Berlin-Frankfurt-Munster; hyper-CVAD: hyper fractionated cyclophosphamide, vincristine, doxorubicin, and dexamethasone.

⁎ Variable treated as continuous.

### Transplant procedure and associated factors

Of 305 patients, 46 (15.1%) underwent HSCT as first-line therapy: 23 (50.0%) received grafts from HLA-haploidentical related donors, 14 (30.4%) from matched related donors, and nine (19.6%) from matched unrelated donors.

A sub-analysis was subsequently performed, focusing on potential candidates for first-line HSCT. In this retrospective cohort, HSCT eligibility was harmonized by selecting transplant candidates who were younger than 65 years, had a Charlson index <2, and exhibited at least one high-risk feature per treatment protocols (Appendix 1). An MRD ≥0.01% on Day +70–78 for those following BFM-like treatment protocols or Day +84 for hyper-CVAD regimen was also considered in candidate selection, in line with current recommendations to incorporate MRD into therapeutic decision-making [5]. Patients with refractory disease, relapse, or early death (<3 months) were excluded (n = 28).

Of 199 potential candidates, 40 (20.1%) underwent first-line HSCT (nine from public and 31 from private healthcare institutions). Among these transplanted patients, the median time from diagnosis to HSCT was 8.4 months (IQR: 6.8–10.3 months) overall: 7.1 months (IQR: 6.7–8.9 months) for HLA-matched related donors, 8.2 months (IQR: 6.9–10.4 months) for haploidentical transplants, and 10.3 months (IQR: 7.1–13.9 months) for matched unrelated donors (p = 0.828). When analyzed by healthcare system, the median time to HSCT was 12.3 months (IQR: 4–23 months) in public versus 7.2 months (IQR: 7.2–35.0 months) in private institutions (p = 0.004).

**Figure 2:**
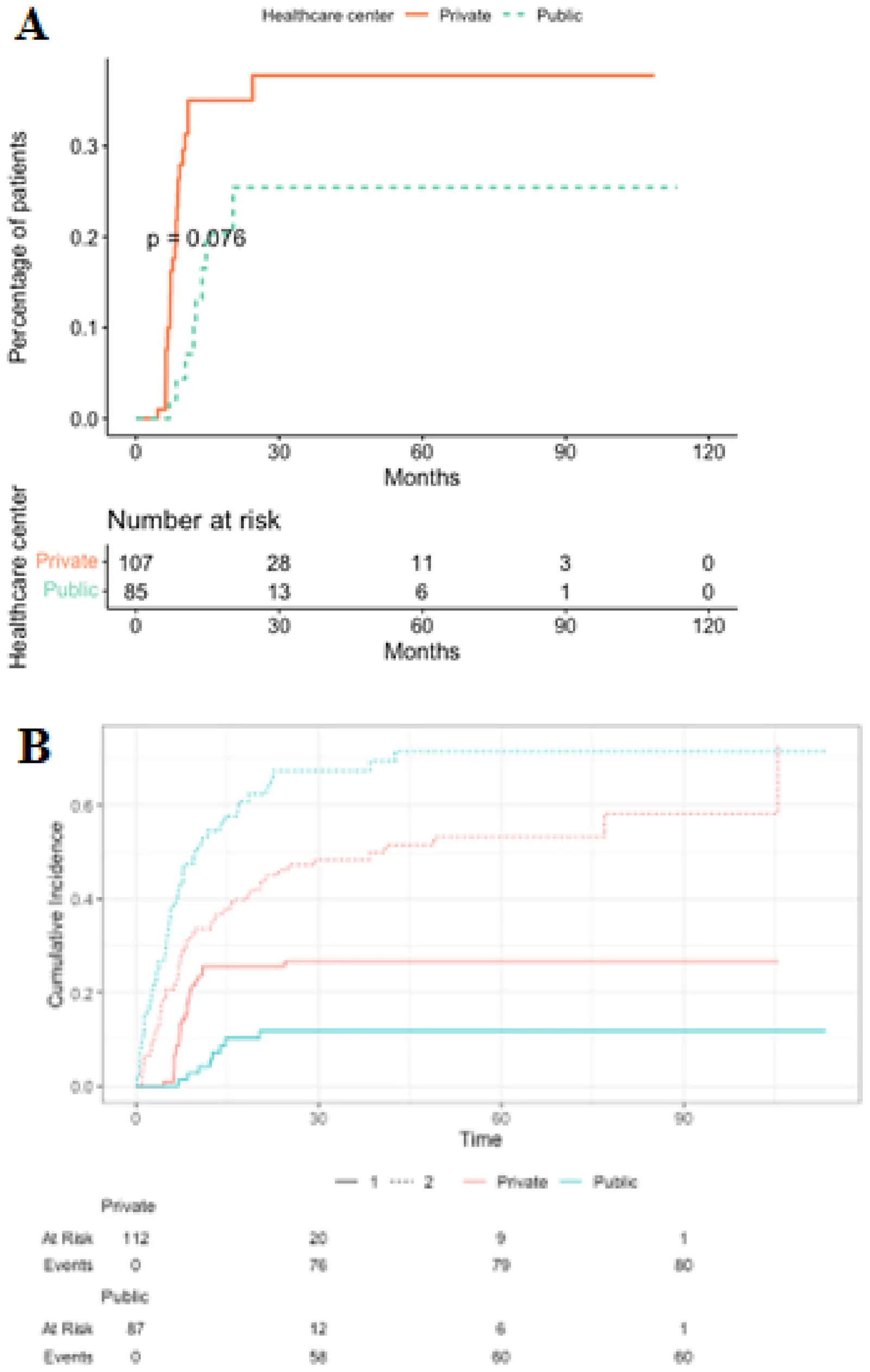
Time to hematopoietic cell transplantation according to healthcare center A) Cumulative Hazard Curve plot; B) Cumulative incidence function considering competing events. 1 = event: transplant; 2 = competing event: death or relapse.

The estimated 1-year cumulative incidence of HSCT, considering relapse and death as competing events, was 16.0% (95% CI: 10.6–21.5). In the multivariate analysis (Table 3), receiving treatment at a public institution was independently associated with a 65% lower likelihood of undergoing HSCT (HR: 0.35; p = 0.013). Similarly, when accounting for death or relapse as competing events, patients from public institutions remained 72% less likely to receive the procedure (Subdistribution HR: 0.28; p = 0.002 - Figure 2). Univariate analyses are provided in Supplementary Table S3.

**Table 3:** Possibility of receiving a transplant (Multivariate analysis).

Covariate	Cox regression model		Fine-Gray model	
	HR (95% CI)	p-value	SHR (97.5% CI)	p-value
Public (vs. private)	0.35 (0.15–0.80)	**0.013**	0.28 (0.12–0.63)	**0.002**
Male (vs. female)	0.69 (0.34–1.39)	0.300	0.70 (0.34–1.44)	0.330
Charlson’s comorbidity index*	0.77 (0.42–1.40)	0.393	0.78 (0.47–1.27)	0.310
High risk (vs. non-high risk)	1.31 (0.28–6.06)	0.732	1.17 (0.28–4.88)	0.830
Age*	0.97 (0.94–1.01)	0.079	0.97 (0.93–1.01)	0.060
Extramedullary involvement -CNS involvement (vs. no involvement) -Other Extramedullary involvement (vs. no involvement)	1.25 (0.59–2.66) 0.41 (0.14–1.25)	0.564 0.118	1.14 (0.53–2.45) 0.47 (0.15–1.43)	0.740 0.180

95% CI: 95% confidence interval; CNS: central nervous system; HR: hazard ratio; SHR: Subdistribution hazard ratio.

⁎ Variable treated as continuous.

## Discussion

Demographic and clinical characteristics as well as response rates and OS of adolescents and adults with ALL in Argentina were similar to those reported in other studies [4,5,13,16,17]. Public funding of the healthcare institution negatively influences OS with a worse outcome when patients were censored at the time of HSCT. Longer time to achieve a first-line HSCT for patients from public healthcare institutions, even when death or relapse were analyzed as competing events, may account for the worse outcome.

The observed median OS of 42 months with 54.9% at two years falls within the range reported by other Latin American groups: around 20 months for Brazilian groups [18] and 56 months for Mexico [19] with similar early death rates [5]. It was confirmed that high-risk, older age and CNS involvement were independently associated with a worse OS in accordance with reported data [16]. Moreover, public funding of the healthcare institution, as an indirect measure of the socioeconomic level, was also an adverse factor. The Argentine healthcare system is highly fragmented; individuals with higher or registered incomes have access to private insurance (either through prepaid financing systems or programs funded by labor contributions), whereas individuals with lower incomes or informal employment receive healthcare from the public sector, which is government-funded. There is vast literature around the world, including Latin America, reporting that socioeconomic level has an impact on the mortality rate [20,21].

Regarding access to appropriate risk stratification, one third of the patients lacked cytogenetic or molecular data, as previously reported in international series [22,23]. This unavailability was greater in resource-limited settings, as previously noticed for adult patients and for those with lower health security coverage [24]. On the other hand, there is broad access to MFC, which helped measure MRD status at different time points, thereby contributing to the individualization of patients with high-risk features.

BFM-like regimens were mostly adopted, and the rate of early death and complete remission (CR) were similar to those previously reported in Latin America [5]. Immunotherapies are increasingly incorporated into frontline treatment of patients with Philadelphia chromosome-positive ALL [25]. The access to Rituximab therapy was similar between public and private institutions, with no registered data on Blinatumomab or Inotuzumab. A national report, however, documented the use of these latter agents in 58 relapsed or refractory cases between 2017 and 2023, with only 3% treated in public institutions [26].

Induction regimens effectively achieve CR, but maintaining long-term remission and curing patients with ALL remains challenging. Eligible high-risk patients may proceed to first-line allo-HSCT with curative intent and to mitigate the risk of relapse. Although a second CR can be achieved in one third of patients, their prognosis is worse. In the present study, 15.1% of the overall cohort and 20.1% of patients deemed to be candidates for allo-HSCT underwent the procedure as a first-line therapy. HSCT rates vary in Latin America from 8% to 32% in different age selected cohorts [27,28]: Ferrari et al. [12] described a rate of 12% among adolescents and young adults in Argentina. Despite the fact that a survey confirms a sustained growth in transplant numbers and rates in the Latin American region between 2019 and 2022 [29], these transplant rates are lower across various diagnoses compared to Europe or the United States [30]. In particular, a Swedish study revealed a significantly higher transplantation rate of 49% among high-risk ALL patients under 60 years of age [31].

Among first-line HSCT recipients in the current cohort, the median time from ALL diagnosis to transplant was 8.4 months. Approximately 20% of the patients received the graft from an unrelated-donor, with a median time that was 10.3 months longer than the 162 days (5.4 months) for unrelated-donor HSCT reported in Canada, a high-income country [32]. This difference may reflect administrative delays and difficulty in finding suitable donors among individuals of South American descent [33]. Basquiera et al. reported a median time to transplant of 18 months for unrelated donor procedures across a cohort encompassing diverse hematological diseases, compared to eight months in the related HLA-matched group [15]. The findings of this study also indicate that being treated in a public healthcare center had a negative impact on the time to HSCT, which is consistent with previous studies from the United States and the United Kingdom [21,34]. Cumulative incidence function curves stratified by healthcare center funding source (Figure 2B) demonstrate a similar early pattern for the competing events of death and relapse, followed by a distinct later divergence in the probability of HSCT.

While the study provides valuable insights, several limitations must be acknowledged mostly in connection with its retrospective nature. Moreover, the limited number of participating centers may not fully capture the clinical landscape across Argentina. Nonetheless, these institutions, where the majority of patients were diagnosed and underwent transplantation, account for one third of all nationally accredited transplant centers.

## Conclusion

This is the first multicenter study from Argentina to analyze demographic and clinical characteristics of adolescent and adult patients with Ph-negative ALL. Overall survival was influenced not only by classical prognostic factors but also by the treatment setting, with patients treated in public institutions having poorer outcomes. Healthcare disparities also influenced the access to first-line HSCT among high-risk patients. Expanding access to transplantation within the public healthcare system may help improve the outcomes and reduce inequities in the management of ALL in Argentina.

## Author contributions

Conceptualization: MMC, JIR, LF, AGC, AD, NC, MMR, MM, CB. Investigation: MMC, JIR, LF, AGC, AD, NC, MMR, MM, CB. Methodology: MMC, JIR, LF, AGC, AD, NC, MMR, MM, CB. Project administration: MMC, JIR, LF, AGC, AD, NC, MMR, MM, CB. Data curation: MMC, JIR, LF, AGC, AD, LR, NC, MMR, EM, LA, CGV, JF, MEF, HDD, MMM, AN, IF, CV, MM, ALB, CB. Formal analysis: MMC, JIR, CB. Software: JIR, CGV, MMM. Resources: JIR, CGV, MMM. Funding acquisition: JIR, CGV, MMM. Writing – original draft: MMC, JIR, LF, AGC, LR, CB. Writing – review & editing: MMC, JIR, LF, AGC, AD, LR, NC, MMR, EM, LA, CGV, JF, MEF, HDD, MMM, AN, IF, CV, MM, ALB, CB. Supervision: MMC, JIR, LF, NC, CV, MM, ALB, CB. Validation: MMC, JIR, LF, NC, CV, MM, ALB, CB. Visualization: MMC, JIR, LF, NC, CV, MM, ALB, CB.

## Ethics statement

This study protocol was reviewed and approved by *Comité de ética independiente del Hospital Alemán* (CEIHA), Hospital Alemán, Buenos Aires, Argentina (CODE 1186, v5.0 [18-NOV-2020]). Informed consent was not obtained. Resolution 1480/2011 of the National Ministry of Health exempts retrospective cohort studies from the obligation to obtain informed consent. The confidentiality of each participant’s identity was adequately preserved according to local regulations (Administración Nacional de Medicamentos, Alimentos y Tecnología [ANMAT]).

## Funding sources

Financial support for this study was provided by 10.13039/100004319Pfizer. The funder had no role in the design, data collection, data analysis, and reporting of this study.

## Data availability

The data that support the findings of this study are available from the corresponding author upon reasonable request.

## Conflicts of interest

CGV is an employee of Pfizer and MMM was an employee of Pfizer at the moment of the study. JIR was an employee of Hospital Alemán, Buenos Aires, Argentina at the moment of the study, which received financial support from Pfizer Inc. The rest of the authors have no conflicts of interest to declare.

## Appendix Supplementary materials

Image, application 1
