# Supplementary material for: Clinical characteristics, prognosis, and achievement of transplant in adolescents and adult patients with Philadelphia chromosome-negative acute lymphoblastic leukemia in Argentina
Source: Hematol Transfus Cell Ther. 2026 Sep 7;48(4):106530. doi: 10.1016/j.htct.2026.106530 (PMC13578365; doi:10.1016/j.htct.2026.106530)
Supplement: Supplementary file 1 [file mmc1.docx]

**Figure S1:** Kaplan-Meier plot for Overall survival.

**Supplementary Material**

**Table S1:** Univariate analysis for overall survival **(n = 305)**

| **Variable** | **n** | **OS (95% CI)** | **Deaths - n** | **HR (95% CI)** | **p-value** |
| --- | --- | --- | --- | --- | --- |
| Healthcare center   - Public - Private | 121  184 | 22 (17-NR)  56 (35-NR) | 59  83 | 1.53 (1.10-2.15) | **0.012** |
| Gender   - Male - Female | 189  116 | 40 (26-61)  49 (31-NR) | 91  51 | 1.12 (0.79-1.58) | 0.521 |
| Extramedullary involvement   - None - CNS involvement - Other extramedullary involvement | 189  59  57 | 42 (28-NR)  17 (11-44)  73 (47-NR) | 86  21  35 | 1.47 (1.04-2.28)  0.68 (0.43-1.10) | **0.032**  0.116 |
| Treatment   - BFM-like - Hyper-CVAD - Not reported | 276  18  11 | 42 (28-67)  35 (15-NR) | 128  9 | 0.91 (0.46-1.79) | 0.789 |
| Risk stratification   - High risk - Non-high risk - Not reported | 146  97  10 | 23 (17-35)  63 (47-NR) | 74  27 | 2.16 (1.47-3.18) | **<0.001** |

95% CI: 95% confidence interval; HR: hazard ratio; CNS: central nervous system; OS: overall survival (months); BFM: Berlin Frankfurt-Munster; hyper-CVAD: hyper fractionated cyclophosphamide, vincristine, doxorubicin, and dexamethasone.

**Table S2.** Baseline characteristics according to healthcare center **(n = 305)**

| **Characteristic** | **n (%)** | **Public, n (%)**  **(N = 121)** | **Private, n (%)**  **(N = 184)** | **p-value** |
| --- | --- | --- | --- | --- |
| Gender   - Male - Female | 189 (62.0)  116 (38.0) | 73 (60.3)  48 (39.7) | 73 (63.0)  68 (37.0) | 0.633* |
| Age (years)   - Median age (IQR) - 18 – 40 - 41-60 - >60 | 32.9 (17.2)  217 (71.1)  65 (21.3)  23 (7.5) | 24.5 (18.9-36.4)  98 (81.0)  20 (16.5)  3 (2.5) | 30.2 (21.6-45.7)  119 (64.7)  45 (24.5)  20 (10.0) | **<0.001#**  **0.002*** |
| Charlson’s comorbidity index   - <2 - ≥2 - Not reported | 269 (88.2)  31 (10.2)  5 (1.6) | 110 (90.9)  6 (5.0)  5 (4.1) | 159 (86.4)  25 (13.6)  9 | **0.019*** |
| CNS involvement   - Yes - No | 59 (19.3)  246 (80.7) | 26 (21.5)  95 (78.5) | 33 (17.9)  151 (82.1) | 0.442* |
| Extramedullary involvement   - Yes - No | 91 (29.8)  214 (70.2) | 31 (25.6)  90 (74.4) | 60 (32.6)  124 (67.4) | 0.192* |
| Treatment   - BFM-like - Hyper-CVAD - Not reported | 276 (90.5)  18 (5.9)  11 (3.6) | 115 (95.8)  5 (4.2)  1 (0.8) | 161 (92.5)  13 (7.5)  10 (5.4) | 0.245* |
| Risk stratification   - High risk - Non-high risk - Not reported | 181 (59.3)  113 (37.0)  11 (3.6) | 76 (65.5)  40 (34.5)  5 (4.1) | 105 (59)  73 (41.0)  6 (3.3) | 0.261* |
| Cause of death   - Infection - Disease progression - Bleeding - Treatment-related toxicity - Not reported | 86 (28.2)  4 (1.3)  6 (2.0)  5 (1.6)  41 (13.4) | 40 (33.1)  4 (3.3)  3 (2.5)  1 (0.8)  11 (9.1) | 46 (25.0)  0 (0.0)  3 (1.6)  4 (2.2)  30 (16.3) | 0.101* |
| Reported karyotype/molecular data:   - Yes - No | 220 (72.1)  85 (27.9) | 76 (62.8)  45 (37.2) | 144 (78.3)  40 (21.7) | **0.003*** |
| Received Rituximab°   - Yes - No | 33  272 | 14 (11.6)  86 (71.1) | 35 (19.0)  149 (81.0) | 0.155 |

CNS: central nervous system; BFM: Berlin-Frankfurt-Munster; hyper-CVAD: hyper fractionated cyclophosphamide, vincristine, doxorubicin, and dexamethasone; IQR: interquartile range.

*Chi2 or Fisher’s exact tests, #Mann-Whitney, ° Patients with CD 20+ status = 73

**Table S3:** Univariate analysis for the possibility of receiving a hematopoietic stem cell transplant (n = 199)

| **Covariate** | **n** | **HSCT** | **Relapse or death** | **Cox regression model** | | | **Fine-Gray model** | | |
| --- | --- | --- | --- | --- | --- | --- | --- | --- | --- |
|  |  |  |  | **Estimated 20-month % cumulative incidence of HSCT (95% CI)** | **HR (95% CI)** | **p-value** | **Estimated 20-month % cumulative incidence of HSCT (95% CI)** | **SHR (97.5% CI)** | **p-value** |
| Healthcare center  - Public  - Private | 87  112 | 9  31 | 53  54 | 18.4 (4.9-30.0)  29.6 (19.2-38.6) | 0.50 (0.22-1.09) | **0.081** | 10.2 (4.4-18.9)  25.6 (17.5-34.4) | 0.36 (0.17-0.77) | **0.008** |
| Gender  - Male  - Female | 125  74 | 24  71 | 30  36 | 20.5 (11.2-28.9)  34.3 (18.8-46.9) | 0.64 (0.33-1.25) | 0.193 | 15.9 (9.7-23.5)  24.4 (14.7-35.3) | 0.70 (0.36-1.34) | 0.280 |
| Extramedullary involvement  - None  - CNS involvement  - Other extramedullary involvement | 121  48  30 | 20  11  4 | 65  27  15 | 26.0 (15.4-35.4)  30.0 (10.9-44.0)  17.8. (1.9-32.4) | 0.71 (0.60-3.01)  1.57 (0.22-1.86) | 0.333  0.406 | 19.2 (12.3-27.3)  21.6 (10.4-35.4)  14.9 (4.5-31.1) | 1.39 (0.68-2.85)  0.75 (0.26-2.19) | 0.370  0.600 |
| Treatment   - BFM-like - Hyper-CVAD - Not reported | 168  12  19 | 37  2 | 84  8 | 27.5 (18.6-35.4)  14.3 (0-36.7) | 0.51 (0.07-3.72) | 0.506 | 27.4 (18.6-36.9)  0 (NC) | 0.46 (0.06-3.45 | 0.450 |
| Risk stratification   - High risk - Non-high risk - Not reported | 173  19  7 | 38  2 | 97  4 | 26.5 (17.9-34.3)  20.0 (0-41.3) | 1.64 (0.39-6.87) | 0.494 | 17.4 (2.4-44.2)  19.8 (1.4-26.5) | 0.63 (0.17-2.27) | 0.480 |
| Charlson's comorbidity index* | _ | _ | _ | - | 0.71 (0.41-1.24) | 0.229 | _ | 0.70 (0.39-1.23) | 0.210 |
| Age* | _ | _ | _ |  | 0.98 (0-96-1.01) | 0.280 | _ | 0.98 (0.95-1.01) | 0.190 |

CI: confidence interval; HR: hazard ratio; SHR: Subdistribution hazard ratio; CNS: central nervous system; BFM: Berlin Frankfurt-Munster; hyper-CVAD: hyper fractionated cyclophosphamide, vincristine, doxorubicin, and dexamethasone; HSCT: hematopoietic stem cell transplant, NC: not calculable.

*Variable treated as continuous

**Appendix 1:** High-risk definition per protocol

**1-Berlin-Frankfurt-Munster (BFM)-like protocols**

a) **PETHEMA protocol** [35]

**Presence of one or more of the following factors:**

- Age between 30 and 55 years. Patients aged 55-60 may be included at the discretion of the treating medical team.
- Leukocyte count > 30×10⁹/L in B-cell precursor ALL.
- Any of the following cytogenetic or molecular abnormalities:
  - Alterations in 11q23 or evidence of MLL rearrangement.
  - Complex karyotype (more than five chromosomal abnormalities).
- Pro-B ALL.
- Pro-T/Pre-T or mature T-cell ALL with any leukocyte count.
- Thymic cortical T-cell ALL with leukocyte count > 100×10⁹/L.

b) **GATLA protocol** [36]

- **Presence of any high-risk cytogenetic-molecular abnormality** (if karyotype is not available, the patient should be stratified according to other prognostic variables):
  - Hypodiploid karyotype.
  - 11q23.3 rearrangements (*KMT2A*, ex-*MLL*).
  - t(9;22), *BCR::ABL1*, Ph(+).
  - Ph-like.
  - Complex karyotype (five or more abnormalities).
  - iAMP21 (*RUNX1* amplifications).
- Poor response to corticosteroids on day 8 (>1,000 blasts/μL in peripheral blood).
- Bone marrow aspirate on day 15: M3 (M3: >20% blasts).
- Multiparametric Flow cytometry (MFC) on day +15: >10% blasts.
- Bone marrow aspirate on day 33: M2 or M3.
- Measurable residual disease (MRD) using MFC on day 33: >0.1% (reassessed post phase 1B of induction, day +78).
- Persistence of extramedullary lesion (testicular, mediastinal, bone) beyond day 33.
- MRD using MFC on day 78: >0.01%

**2-Hyper-CVAD** [37]

Adverse risk factors:

- Age ≥ 45 years
- Leukocytosis ≥50 x > 100×10⁹/L
- Poor performance status (an Eastern Cooperative Oncology Group score of 3-4)
- Ph-positive disease
- French-American-British L2 morphology
- >1 course to achieve complete remission (CR)
- Day 14 bone marrow blasts > 5%.

Patients were divided into low-risk (risk score 0-1), intermediate risk (risk score 2-3), and poor-risk groups (risk score ≥ 4).
